# An Interface ASIC for MEMS Vibratory Gyroscopes with Nonlinear Driving Control

**DOI:** 10.3390/mi10040270

**Published:** 2019-04-22

**Authors:** Risheng Lv, Qiang Fu, Liang Yin, Yuan Gao, Wei Bai, Wenbo Zhang, Yufeng Zhang, Weiping Chen, Xiaowei Liu

**Affiliations:** 1 MEMS Center, Harbin Institute of Technology, Harbin 150001, China; lvrisheng@hit.edu.cn (R.L.); qiangfu.hit@gmail.com (Q.F.); 17S021055@stu.hit.edu.cn (Y.G.); 17S021061@stu.hit.edu.cn (W.B.); 17B321006@stu.hit.edu.cn (W.Z.); yufengzhang.hit@gmail.com (Y.Z.); weipingchen.hit@gmail.com (W.C.); 2 Key Laboratory of Micro-Systems and Micro-Structures Manufacturing (Harbin Institute of Technology), Ministry of Education, Harbin 150001, China; 3 State Key Laboratory of Urban Water Resource & Environment, Harbin Institute of Technology, Harbin 150001, China

**Keywords:** micro-electromechanical systems (MEMS) vibratory gyroscope, nonlinear multiplier, automatic gain control, incremental zoom analog-to-digital converter (ADC)

## Abstract

This paper proposes an interface application-specific-integrated-circuit (ASIC) for micro-electromechanical systems (MEMS) vibratory gyroscopes. A closed self-excited drive loop is employed for automatic amplitude stabilization based on peak detection and proportion-integration (PI) controller. A nonlinear multiplier terminating the drive loop is designed for rapid resonance oscillation and linearity improvement. Capacitance variation induced by mechanical motion is detected by a differential charge amplifier in sense mode. After phase demodulation and low-pass filtering an analog signal indicating the input angular velocity is obtained. Non-idealities are further suppressed by on-chip temperature drift calibration. In order for better compatibility with digital circuitry systems, a low passband incremental zoom sigma-delta (ΣΔ) analog-to-digital converter (ADC) is implemented for digital output. Manufactured in a standard 0.35 μm complementary metal-oxide-semiconductor (CMOS) technology, the whole interface occupies an active area of 3.2 mm^2^. Experimental results show a bias instability of 2.2 °/h and a nonlinearity of 0.016% over the full-scale range.

## 1. Introduction

Over the last decade, micro-electromechanical system (MEMS) based inertial sensors have found widespread developments in both research efforts and commercial products owing to their preponderance of low cost, small size, low power consumption and suitability for batch fabrication, and are consequently extensively used for detecting angular rates and accelerations in all spatial directions [1,2,3,4]. As consumer devices, these types of sensors are mostly applied for driving assistance and indoor navigation. With the incorporation of gyroscopes, accelerometers, and magnetic sensors, an inertial measurement unit (IMU) is constituted and nearly essential in the field of independent navigation improvement especially when satellite navigation is not available [5,6]. MEMS gyroscopes, as one of the most widely used inertial sensors, have irreplaceable application requirements and significant research value. Recently, rapid development and mass utilization have raised heated interests in both micromechanical sensitive structures and corresponding interface circuits [7,8]. Despite diverse designs of MEMS gyroscopes based on variant principles, vibratory rate gyroscopes are the most investigated [9]. Regardless of practical materials and structures, hemispherical, disc, and ring resonating gyroscopes are based on a fundamental Coriolis effect without any exceptions [10,11,12,13,14,15]. Generally, the structure of a typical micromechanical vibratory rate gyroscope has at least 2-degree-of-freedom (DOF) motion capability in orthogonal directions to achieve a Coriolis induced energy transfer between two separate resonance modes (drive and sense), so that two corresponding controlling loops are demanded in collocated interface application-specific-integrated-circuits (ASICs). The scheme of the closed drive loop is diffusely employed and researched in published literature and commercial products. Stable and accurate driving control guarantees preconcerted mechanical motion of the central proof mass and rigorous demodulation reference for sense loop [16]. Furthermore, driving accuracy involves low-distortion electrostatic actuation. The startup rate is another major criterion. Consequently, a conjoint driving design is a valuable method.

A key technique proposed in this work is the design of a nonlinear multiplier for hybrid driving control. Especially, a full-scale square-wave is generated as differential electrostatic driving forces for rapid oscillation during incipient electrification. Smooth transition to a couple of stable sinusoidal signals is realized afterward. As a result, resonance speed and precise driving are compatible.

In this paper, an interface ASIC for MEMS vibratory gyroscopes with an original method of nonlinear driving control is presented. A PI-controller is employed in the drive loop for maintaining stability and automatic control. The reliable scheme of switch demodulation is implemented in a sense loop. An incremental zoom analog-to-digital converter (ADC) is adopted for the availability of on-chip temperature calibration as well. Manufactured in standard 0.35 μm complementary metal-oxide-semiconductor (CMOS) technology provided by HHGrace (Shanghai, China), this work achieves a bias instability of 2.2 °/h with a full-scale range of ±400 °/s and dissipates 10.2 mW.

This paper is organized as follows. Section 2 illustrates the mechanical vibration modeling. System description and implementation details are adequately described in Section 3 and Section 4, respectively. Experimental results are shown and discussed in Section 5, and the conclusions are finally drawn in Section 6.

## 2. Mechanical Vibration Modeling and Calculation

### 2.1. Mechanical Model Analysis

Angular velocity is generally detected in micromechanical vibratory gyroscopes based on the Coriolis effect. A proof mass and corresponding connected frames in the MEMS process are essential and used to realize motion detection. When the proof mass is driven by electrostatic forces and maintains a resonance state, an externally perpendicular angular velocity will trigger a tangential Coriolis force resulting in a relevant forced vibration. Variation of relative positions between the fingers connected to the proof mass and ambient frames caused by the vibration in sense direction induces capacitance diversification, from which the interface ASIC calculates motion characteristics and accomplish angular velocity detection.

**Figure 1:**
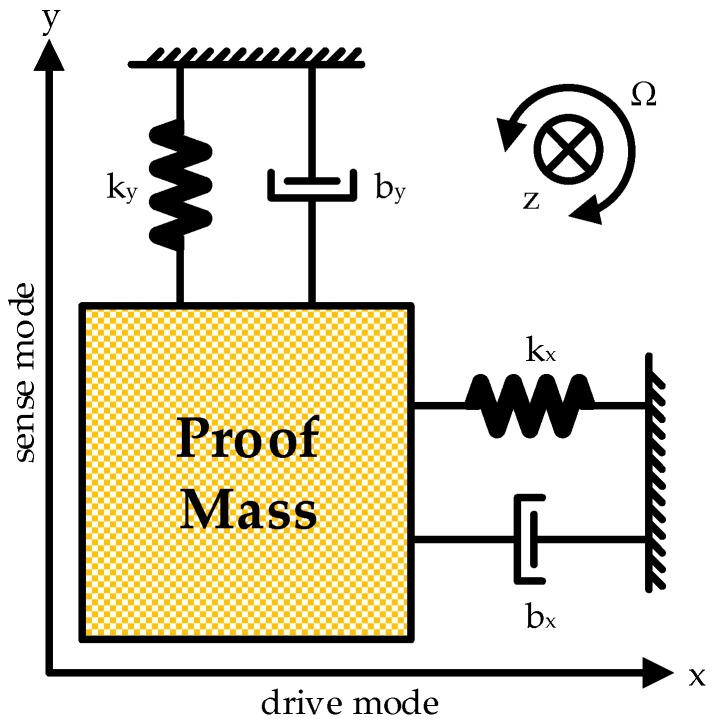
Mechanical vibration model for MEMS gyroscopes.

The operating principle is briefly described by a spring oscillator model, as shown in Figure 1. The proof mass is connected to the external frame by independent springs. Specifically, *k_x_* and *k_y_* denote the stiffness coefficients of the two connecting springs, *b_x_* and *b_y_* the damping coefficients, respectively. Simple harmonic vibration will be obtained in the drive axis (x-direction) when the proof mass is driven by an electrostatic force *F_d_* with the same frequency as its natural drive frequency *ω_d_*. Creation conditions of Coriolis force in a non-inertial reference system are therefore satisfied if an angular velocity Ω emerges along z-direction.

Concretely, the proof mass is dominated by an externally driving electrostatic force *F_d_* as well as elastic and damping forces in connected springs. When an angular velocity Ω in *z* axis appears, physical motion of the proof mass is described by

(1) $$F_{d}-b_{x}\dot{x}-k_{x}x=m\ddot{x}-m\Omega^{2}x-2m\Omega \dot{y}-m\dot{\Omega }y,$$

where *x* and *y* express the drive and sense displacement in corresponding directions, respectively. Since the resonance displacement *y* in sense direction is relatively small, $m\dot{\Omega }y$ in (1) can be therefore omitted. It is also necessary to be noted that the coupling Coriolis force $2m\Omega \dot{y}$ is insignificant compared to *F_d_*. Also, $m\Omega^{2}x$ is ignored, since $m\Omega^{2}x<<k_{x}$. The final simplified equation is consequently calculated as

(2) $$m\ddot{x}+b_{x}\dot{x}+k_{x}x=F_{d}.$$

There is, however, no external driving force in the sense mode. As a result, the Coriolis force induced by simple harmonic vibration in the x-direction acts as the dominant element and cannot be ignored. Similarly, the final simplified equation in sense mode is

(3) $$m\ddot{y}+b_{y}\dot{y}+k_{y}y=-2m\Omega \dot{x}.$$

### 2.2. Mechanical Motion Regulation

**Figure 2:**
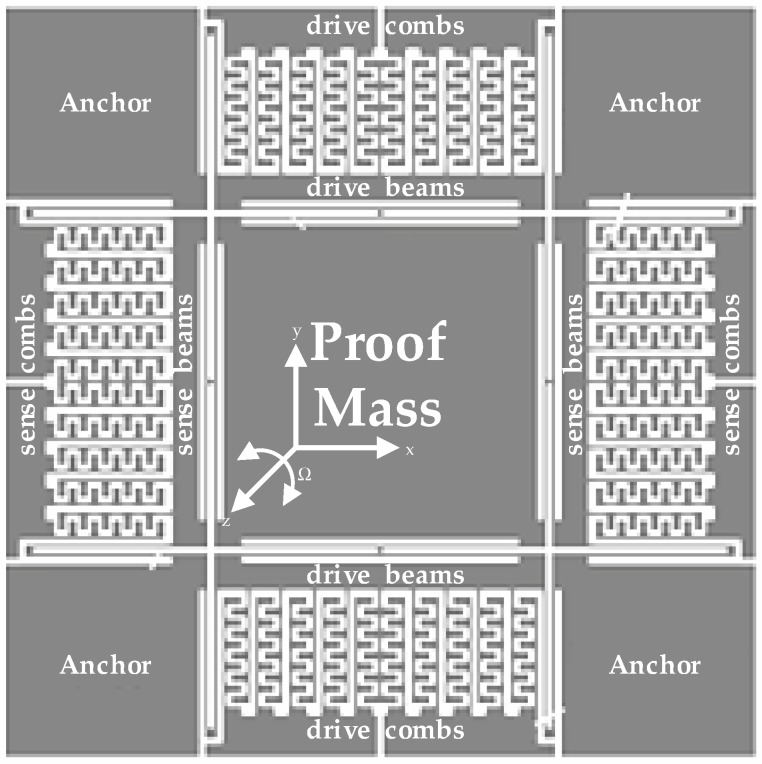
A standard model of the sensitive structure for MEMS gyroscopes.

MEMS vibratory gyroscopes are composed generally of a proof mass with elastic beams and corresponding combs in both directions for capacitance detection in ASIC interfaces. Figure 2 plots a standard model of the sensitive structure for MEMS gyroscopes with a single proof mass driven in the x-direction. The sensitive orientation is therefore vertical to the manufactured plane, namely the z-direction. This gyroscope employs a symmetrical design, in which a vibratory proof mass is in the center, and four anchors in corners are used for fixation. Both drive and sense combs are connected indirectly to the central proof mass through each internal elastic beam. Sensitive structures are usually packaged in vacuum cavities for lower Brawnian noise according to actual application requirements. If an alternating voltage is loaded on capacitors between fixed and free combs in the drive axis, a relevant electrostatic force drives the free combs into vibration. When this driving force shares the same frequency as the natural frequency, resonance is realized as a precondition for Coriolis effect. In the event of an angular velocity Ω in the z-direction, a corresponding Coriolis force drives the proof mass into a state of forced vibration. As a result, capacitances between sense combs vary, and the differences are in proportion to Ω. The initial angular velocity Ω is consequently obtained through capacitance measurement, and gyroscopes are realized. It is also evident that detection precision is closely related to capacitance variation between combs.

**Figure 3:**
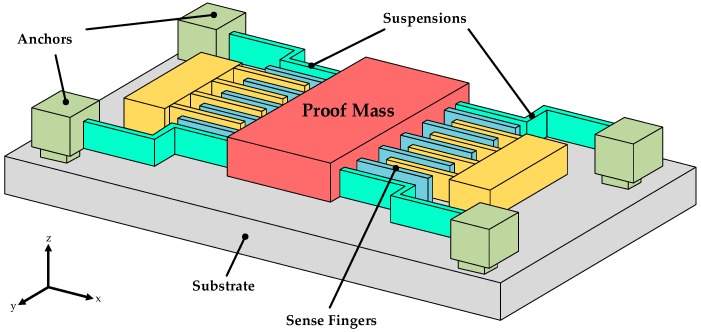
Schematic diagram of driving fingers.

Based on the above illustration, the electrical properties of electrostatic driving are calculated from a simplified universal model for micromechanical inertial sensors in Figure 3 [17].

In order for explicit expressions, use *M_d_* for the equivalent mass in drive axis, and *ω_d_* for the natural frequency of drive mode, *ξ_d_* for damping ratio, *Q_d_* for the overall systematic quality factor. Generally, the driving displacement *x*(*t*) follows

(4) $$x(t)=B_{d}\sin(\omega t+\varphi_{d}),$$

where $B_{d}=F_{o}/M_{d}{\omega_{d}}^{2}\sqrt{{\left(1-\frac{\omega^{2}}{{\omega_{d}}^{2}}\right)}^{2}+{\left(\frac{\omega }{Q_{d}\omega_{d}}\right)}^{2}}$, $\varphi_{d}=\arctan\frac{-2\xi_{d}\omega_{d}\omega }{{\omega_{d}}^{2}-\omega^{2}}$.

The motion features are similar in the sense axis. Based on the universal mechanical analysis, the total motion displacement *y*(*t*) can be expressed as

(5) $$\begin{array}{l} y(t)=(B_{s1}+B_{s2})\cos(\omega_{i}t+\frac{\varphi_{s2}-\varphi_{s1}}{2})\cos(\omega t+\frac{\varphi_{s1}+\varphi_{s2}}{2}+\varphi_{d})\\ +(B_{s1}-B_{s2})\sin(\omega_{i}t+\frac{\varphi_{s2}-\varphi_{s1}}{2})\sin(\omega t+\frac{\varphi_{s1}+\varphi_{s2}}{2}+\varphi_{d}) \end{array}$$

It is clear that the remaining two terms are mutually orthogonal, and that the first term domains the amplitude of *y*(*t*) and is a suitable candidate for final output. Choose $\cos(\omega t+\frac{\varphi_{s1}+\varphi_{s2}}{2}+\varphi_{d})$ for demodulation and we get the final output after filtering as

(6) $$y(t)=\frac{1}{2}(B_{s1}+B_{s2})\cos(\omega_{i}t+\frac{\varphi_{s2}-\varphi_{s1}}{2}).$$

The systematic output is characteristic of the same frequency as input angular velocity and a proportional amplitude with it and is therefore measured for angular velocity detection.

## 3. System Description and Topology Analysis

**Figure 4:**
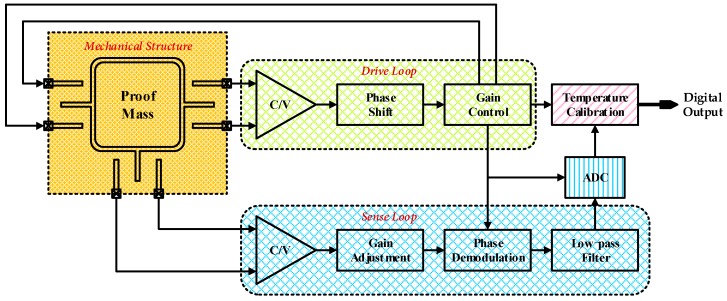
Systematic design of the proposed interface for MEMS gyroscopes.

The proposed interface for ASIC for MEMS vibratory gyroscopes is composed of a self-excited closed drive loop as well as an open loop for angular velocity detection with additional ADC and on-chip temperature calibration, as shown in Figure 4.

**Figure 5:**
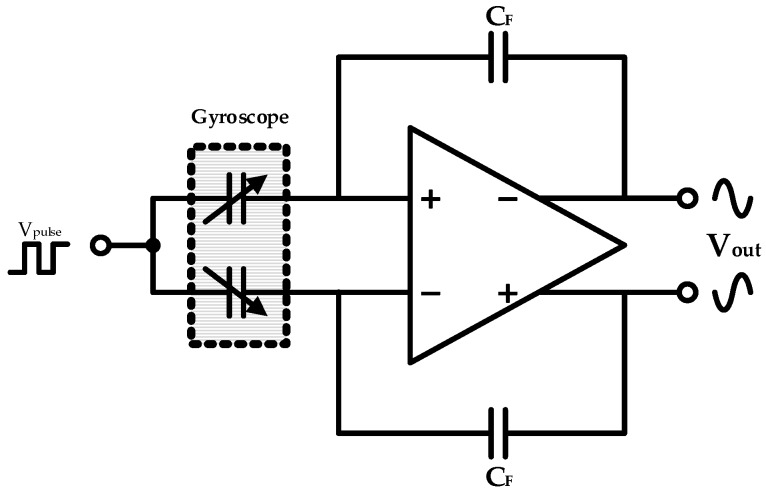
C/V conversion stage in gyroscope interface ASICs.

The employed drive loop consists mainly of a differential C/V conversion module for capacitance detection and a section of gain control. Specifically, the capacitors between combs and analog front-end stage circuit (operational amplifier and feedback capacitors) compose the differential charge amplifier for C/V conversion, as shown in Figure 5. The following element is an amplitude controlling segment based on PI control. Moreover, the proposed nonlinear driving control is accomplished here by a novel nonlinear multiplier illustrated later in detail. A pair of differential voltage outputs return then back to the driving combs of the whole sensitive mechanical structure for electrostatic actuation, thus constituting a closed-loop driving control.

On the other hand, the interface for sense mode begins likewise with a similar differential C/V conversion module. The dominant portion in this mode is a procedure of phase demodulation for input angular velocity resolving from detection voltage signals. A switch phase sensitivity demodulation is employed in this work. The stable signal in the drive loop is used here as the demodulation signal. After removal of high-frequency interferences by a low-pass filter, a final output is obtained for representation of initial input angular velocity. A high-precision analog-to-digital converter (ADC) is also employed for better compatibility across analog and digital application systems as well as easy access to on-chip digital operation of non-idealities suppression. The overall system ends up with a temperature calibration module for the elimination of temperature drift in both mechanical sensitive structure and corresponding interface ASIC.

### 3.1. Research on Self-Excited Drive Loop

According to the principles and analyses of the proposed drive loop above, a couple of differential alternating electrical signals are loaded between symmetrical electrodes of free combs and the central proof mass. When the Barkhausen’s criteria is satisfied as expressed in (7), self-excitation is achieved with the aid of favorable mechanical frequency-selection characteristics.

(7) $$\left\{ \begin{array}{l} \left|\beta H(j\omega_{0})\right|=1\\ ∠\beta H(j\omega_{0})=-180^{\circ } \end{array}\right.$$

On the assumption of differential alternating electrical signals $V_{dc}\pm V_{ac}\sin\omega t$, the vibration displacement of the proof mass is

(8) $$x(t)=\left[4N_{1}\varepsilon \frac{z}{y}V_{dc}V_{ac}/M_{d}{\omega_{d}}^{2}\sqrt{{\left(1-\frac{\omega^{2}}{{\omega_{d}}^{2}}\right)}^{2}+{\left(\frac{\omega }{Q_{d}\omega_{d}}\right)}^{2}}\right]\sin(\omega t+\arctan\frac{-2\xi_{d}\omega_{d}\omega }{{\omega_{d}}^{2}-\omega^{2}}),$$

according to (4), where *N_1_* denotes the number of driving electrodes and *ε* the vacuum permittivity. On the condition of a self-excited closed loop, the frequency of alternating drive voltage *ω* automatically tracks the natural frequency of drive mode *ω_d_*. Since the damping ratio $\xi_{d}=1/2Q_{d}$, (8) is simplified as

(9) $$x(t)=-\left[2N_{1}\varepsilon \frac{z}{y}V_{dc}V_{ac}/M_{d}{\omega_{d}}^{2}\xi_{d}\right]\cos(\omega_{d}t).$$

When a DC voltage *V_p_* is loaded on the central proof mass, the corresponding current induced by vibration in drive axis through capacitors comprised of fingers is

(10) $$i_{0}=\frac{dQ}{dt}=\frac{d(CV_{p})}{dt}=C\frac{dV_{p}}{dt}+V_{p}\frac{dC}{dt}=V_{p}\frac{dC}{dt}.$$

Supposing there are *N_2_* sense fingers in drive axis, the total current is calculated as

(11) $$i=N_{2}V_{p}\frac{dC}{dt}=N_{2}V_{p}\frac{dC}{dx}\frac{dx}{dt}=N_{2}V_{p}\varepsilon \frac{z}{y}\frac{dx}{dt}.$$

Based on (9), (11) is rearranged as

(12) $$i=N_{2}V_{p}\varepsilon \frac{z}{y}\frac{dx}{dt}=\frac{2N_{1}N_{2}\varepsilon^{2}z^{2}V_{p}V_{dc}V_{ac}}{y^{2}M_{d}{\omega_{d}}^{2}\xi_{d}}\sin(\omega_{d}t).$$

The overall closed-loop gain maintains unit by adjusting latter gain-control module, and therefore a closed drive loop is obtained. Supposing the equivalent gain of the automatic gain control is *G*, the driving signal equals

(13) $$V_{drive}=-\frac{2N_{1}N_{2}\varepsilon^{2}z^{2}V_{p}V_{dc}V_{ac}}{C_{f}y^{2}M_{d}{\omega_{d}}^{2}\xi_{d}}G\sin(\omega_{d}t).$$

Since the differential voltages loaded on driving combs follow $V_{dc}\pm V_{ac}\sin\omega t$, a self-excited closed drive loop is realized on condition that

(14) $$\frac{2N_{1}N_{2}\varepsilon^{2}z^{2}V_{p}G}{C_{f}y^{2}M_{d}{\omega_{d}}^{2}\xi_{d}}=1.$$

### 3.2. Research on Precise Sense Loop

According to the principal illustration of the mechanical structure, a similar vibration in sense axis occurs in case of input angular velocity. A couple of symmetrical capacitances are

(15) $$C_{s1}=\frac{\varepsilon xz}{d_{0}-y},C_{s2}=\frac{\varepsilon xz}{d_{0}+y}.$$

Assume the number of the sense fingers in sense axis is *N*_3_ and that the feedback capacitance in the differential charge amplifier is *C_f_*, the output voltage after C/V conversion is

(16) $$V_{sense}=N_{3}\frac{C_{s1}-C_{s2}}{C_{f}}V_{p}\approx \frac{2\varepsilon xyzN_{3}}{{d_{0}}^{2}C_{f}}V_{p}.$$

As mentioned above, choose a square wave with an amplitude of *V_c_* derived from drive loop for demodulation, a final low-frequency output carrying the initial input angular velocity is obtained after filtering is calculated as

(17) $$V_{out}=\frac{4\varepsilon xzN_{3}}{\pi {d_{0}}^{2}C_{f}}V_{p}V_{c}(B_{s1}+B_{s2})\cos(\frac{\varphi_{s1}+\varphi_{s2}}{2})\cos(\omega_{i}t+\frac{\varphi_{s1}-\varphi_{s2}}{2}).$$

It is clear that all necessary information is included in (17). Also, overall systematic sensitivity depends on the amplitude and response speed on the phase.

## 4. Circuit Implementation Details

### 4.1. Nonlinear Multiplier

Apart from the reduction of systematic thermal noise and flicker noise, distortional elements in drive signals are fatal for high-precision gyroscopes. The central proof mass is driven by either square-wave or a sinusoidal signal in conventional schemes. Driving signals are adjusted in amplitudes by the PI controller. Generally, the scheme of square-wave driving stands out in simple implementation and rapid oscillation, and sinusoidal driving in satisfying accuracy. Both methods, however, have their own drawbacks. Specifically, many distortional components of harmonics are simultaneously contained in a square wave which leads to phase noise and consequent interferences for demodulation in sense loop. Despite the simplex frequency constituent, a sinusoidal driving signal cannot provide adequate gain initially so that a stable driving resonance needs several dozens of seconds at least.

This work employs a fixed DC voltage as a partial driving element and alters electrostatic driving force on proof mass by adjusting the amplitude of combined AC voltage. The implemented nonlinear adjustment is practically realized by the proposed nonlinear multiplier. During the period of resonance establishment, a strong driving force is fulfilled for rapid oscillation. Dynamic and stable balance is also achieved afterward. The gain of the nonlinear multiplier is sufficiently large at the beginning and turns to the approximate unit after stabilization.

**Figure 6:**
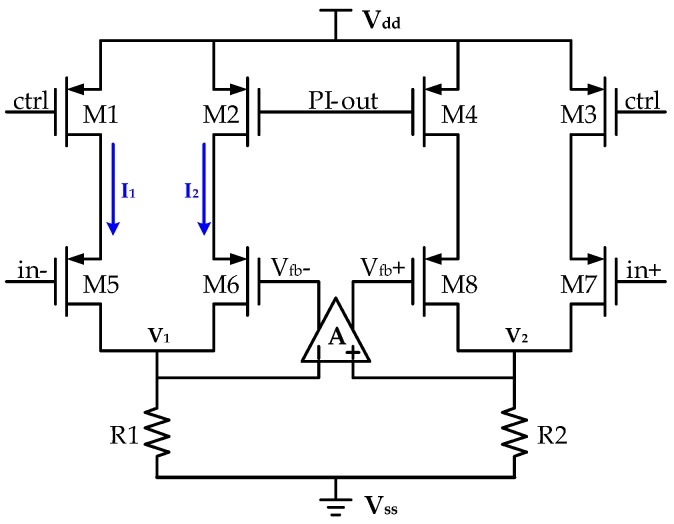
Schematic of the proposed nonlinear multiplier.

The proposed nonlinear multiplier is provided in Figure 6. When the whole interface circuit starts, there is no resonant signal in the drive loop. At this moment, the former PI controller gives a saturated voltage (*V_dd_*). Hence M2 and M4 pinch off. The internal assistant amplifier *A* consequently operates at an open-loop stage, and the corresponding infinite gain results in saturated outputs (*V_fb_*+ and *V_fb_*−). In consequence, a square-wave driving is realized initially. On account of the mechanical frequency-selection characteristic, the driving signal increases gradually and the PI controller generates a larger output voltage. The currents in the four paths tend to be equal and finally form a dynamic balance.

The proposed multiplier is designed in the differential and symmetrical structure for harmonic suppression, as shown in Figure 6. Four inputs and two outputs are included. A couple of differential driving signals in the drive loop (in+ and in-) are two of the input ports. The output of the PI controller (PI-out) and a gain controlling port (ctrl) are the other two. A couple of differential sinusoidal outputs (*V_fb_*+ and *V_fb_*−) are adopted as outputs of the multiplier. A branch circuit composed of M1 and M5 is referred to as a common-source stage in the triode region, and the equivalent transconductance *G*_m1_ is

(18) $$G_{m1}=\mu_{p}C_{ox}{\left(\frac{W}{L}\right)}_{1}\left(V_{dd}-V_{ctrl}-V_{TH}\right).$$

And the corresponding circuit in this branch is

(19) $$I_{1}=G_{m1}\cdot V_{in-}.$$

Supposing the gain of the embedded operational amplifier is *A*, the gate voltage of M6 is

(20) $$V_{fb^{-}}=AV_{1}=2AI_{1}R_{1}.$$

The equivalent transconductance *G*_m2_ is similarly expressed as

(21) $$G_{m2}=\mu_{p}C_{ox}{\left(\frac{W}{L}\right)}_{2}\left(V_{dd}-V_{PI-out}-V_{TH}\right).$$

On account of the employed negative feedback, a dynamic balance is obtained between I_1_ and I_2_, in other words I_1_ = I_2_. Then *V_fb_*− is rewritten as

(22) $$V_{fb^{-}}=\frac{G_{m1}}{G_{m2}}V_{in-}=\frac{V_{dd}-V_{ctrl}-V_{TH}}{V_{dd}-V_{PI-out}-V_{TH}}V_{in-}.$$

In this way, the overall gain of the multiplier is adjusted by the PI controller. An automatic gain controlling is therefore achieved and the closed drive loop is stabilized.

Given the nonlinear characteristics of metal oxide semiconductor (MOS) transistor, high-order distortion in each branch circuit is simultaneously subsistent. Take I_1_ as an example,

(23) $$I_{1}=\alpha_{1}V_{in-}+\alpha_{2}V_{in-}^{2}+\cdot \cdot \cdot +\alpha_{n}V_{in-}^{n},$$

where α_i_(i = 1,2,…,n) is corresponding scale factor. Components higher than 3rd-order are omitted without loss of generality. In consequence, these distortion terms are induced into the output *V_fb_*−, as illustrated in (24).

(24) $$V_{fb^{-}}\approx k_{1}V_{in-}+k_{2}V_{in-}^{2},$$

where *k*_i_ = α_i_/*G*_m2_(i = 1,2,…,n). Similarly, we have

(25) $$V_{fb^{+}}\approx k_{1}V_{in+}+k_{2}V_{in+}^{2}=-k_{1}V_{in-}+k_{2}V_{in-}^{2}.$$

The final acting driving signal is the difference between *V_fb_*+ and *V_fb_*− and is calculated as

(26) $$V_{drive}=\left|V_{fb^{+}}-V_{fb^{-}}\right|=2k_{1}V_{in-}.$$

Even-order harmonic distortion is therefore absolutely eliminated theoretically.

**Figure 7:**
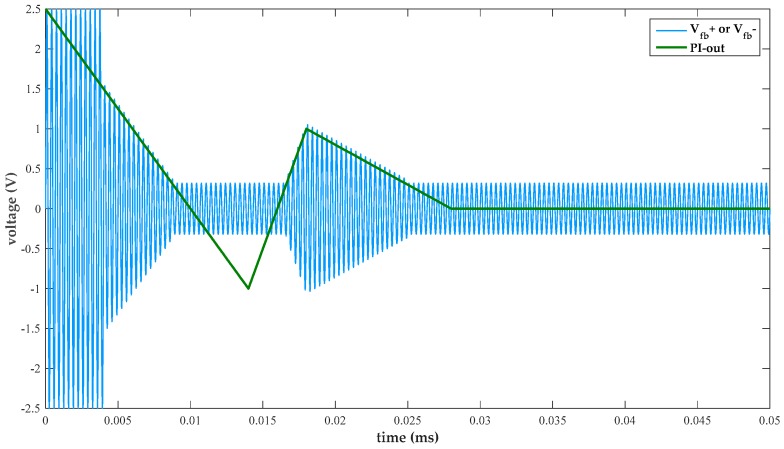
Transient simulation of the proposed nonlinear multiplier.

Figure 7 plots a transient simulation results of the proposed nonlinear multiplier. When the differential inputs (in+ and in-) are fixed, differential outputs vary with PI-out as shown in Figure 7. It is apparent that differential outputs are controlled by PI-out, and that a smooth transition from a square wave to sinusoidal signal is achieved.

**Figure 8:**
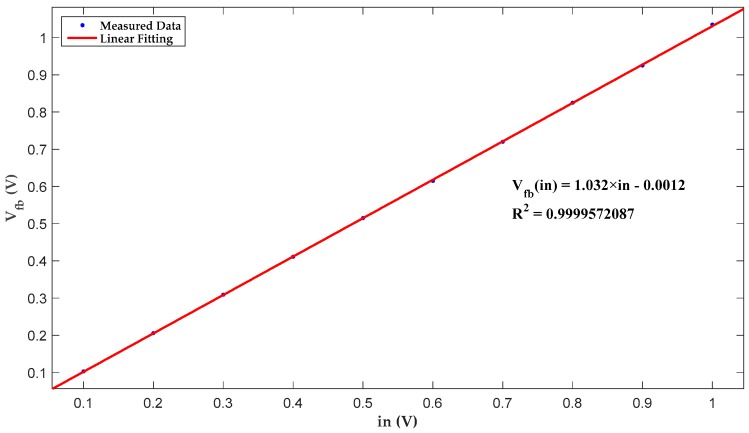
Linear fitting of multiplier simulation results.

A group of simulation results in Table 1 reveals a nonlinearity of 0.2168% so that low-distortion driving is obtained. The corresponding fitting curve is plotted in Figure 8.

### 4.2. Incremental Zoom ADC

**Figure 9:**
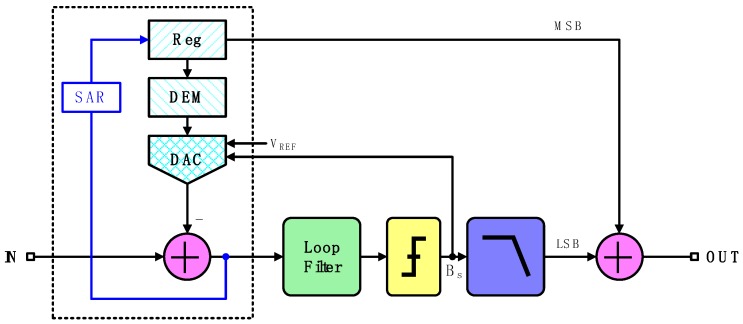
Block diagram of the employed zoom analog-to-digital converter (ADC).

Low conversion bandwidth is characteristic of a high-precision zoom ADC, which happens to suit for digitalization of the final output in a MEMS vibratory gyroscope with a usual bandwidth of several tens of Hertz. Figure 9 gives a block diagram of a zoom incremental ADC. Combined with both successive approximation register (SAR) and Sigma-Delta (ΣΔ) structures, this ADC realizes high precision without an extremely high over-sampling ratio (OSR) [18]. Especially, analog voltage signals indicating angular velocity are preliminarily converted by a SAR ADC to realize coarse conversion. This work employs a 6-bit SAR ADC for hardware consumption economy and rapid reference interval confirmation to provide for subsequent ΣΔ conversion, during which further process of oversampling and noise shaping is accomplished [19]. Furthermore, the elementarily locked references allow a single-bit quantizer which is equipped with inherent linearity [20]. Besides, the quantizer output is scaled independently of the output of the preceding loop filter. Quantization error is therefore largely suppressed and nonlinearity unavoidably induced by a multi-bit quantizer is evaded as well. The employment of the dynamic element matching (DEM) technique also helps restrain capacitance mismatch.

This combination of both structures coalesce the advantages of speed efficiency of a SAR ADC and high precision of a ΣΔ ADC. As a result, merely medium-precision ADCs are needed for accurate data conversion. It is obvious that harsh terms for corresponding circuits are no more compulsive and that lower power dissipation and device economy are simultaneously achieved.

**Figure 10:**
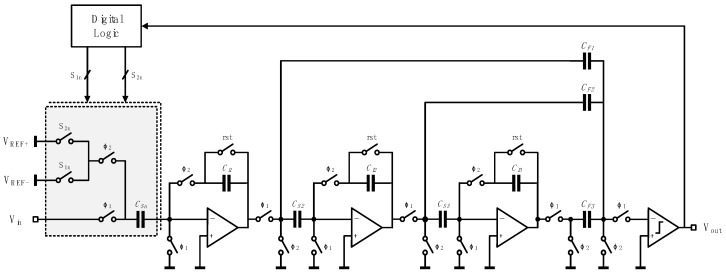
Simplified schematic of the analog modulator.

A simplified schematic of the analog modulator in zoom ADC is shown in Figure 10. Cooperating with a coarse 6-bit SAR ADC, only three stages in the ΣΔ ADC are needed for an effective number of bits (ENOB) of 24.

## 5. Experimental Results and Discussion

**Figure 11:**
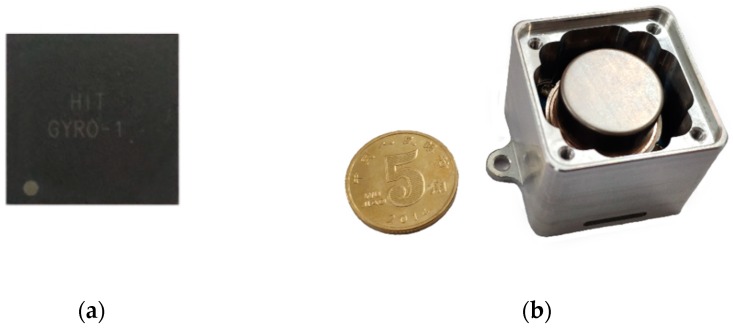
(**a**) Packaged ASIC. (**b**) Prototype gyroscope.

The proposed interface ASIC for MEMS vibratory gyroscopes was designed and fabricated in a standard 0.35 μm CMOS technology. This ASIC was tested in coordination with a vacuum packaged MEMS sensing element provided by Peking University (Beijing, China). The typical parameters include a resonance frequency of 6 kHz and a high-quality factor (Q) of approximate 10^4^ [21]. The packaged chip is shown in Figure 11a. Figure 11b plots the prototype entire gyroscope including the implemented ASIC, the MEMS sensing element, and the testing printed-circuit-board (PCB). The MEMS sensing element is sealed in a vacuum metal package and bonded to this PCB. Operating at normal temperature, the whole interface dissipates 10.2 mW from a 5 V supply.

**Figure 12:**
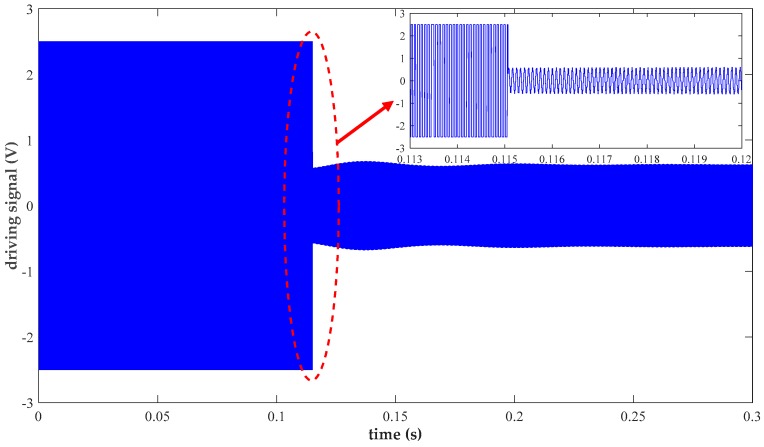
Measurement of the nonlinear drive loop.

The electrification stage of the drive loop is first tested to ensure a reliable resonance oscillation in the central proof mass as the essential precondition for the Coriolis effect. Figure 12 shows the startup process of the self-excited closed drive loop. The complete changing process of the practical driving signal is well explicitly clarified. Specifically, rapid square-wave driving is initially obtained to minimize the starting time. Next, the fixed amplitude of the sinusoidal driving signal indicates the establishment of final stable resonance within 200 ms. Harmonic distortion is adequately suppressed due to the proposed method of nonlinear driving control. Experimental results measured by a dynamic analyzer HP35670 reveal a distortion factor of 1.056%, which largely weakens its adverse impact on precise angular velocity resolving.

**Figure 13:**
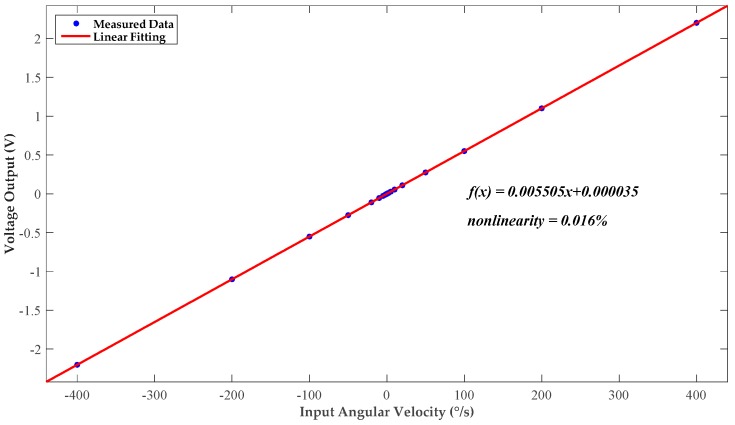
DC transfer function after calibration.

Typical values of input angular velocity are selected within the regulation range and applied during the test. An average value of 100 measurement results is recorded as the corresponding output for each test point. Figure 13 plots the calibrated measurement voltage output as a function of various input angular velocities at discriminative values. The result of linear fitting shows a scale factor of 5.5 mV/°/s and a zero-rate output of 35 μV. The fitting nonlinearity over the measurement range reaches up to 0.016%.

**Figure 14:**
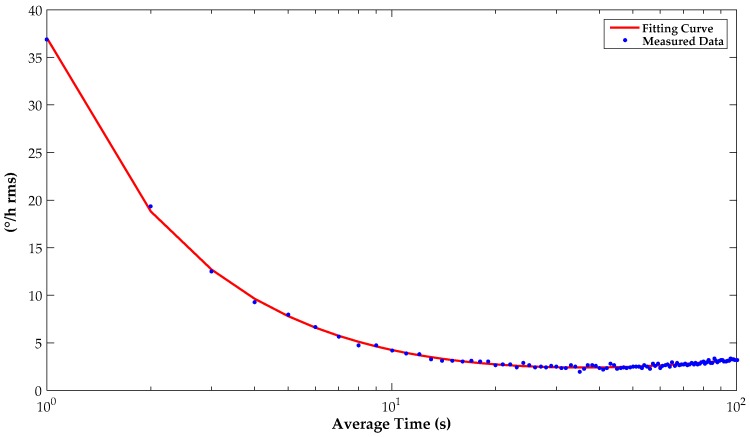
Standard Allan variance at 25 °C.

The overall gyroscope is tested on a settled rate table in an incubator which is set at a constant normal temperature to avoid interferences induced by environmental vibration and temperature drift. Figure 14 shows the Allan variance of the proposed gyroscope which identifies various systematic noise sources and determines long-term stability. The bias instability of 2.2 °/h shown in the experimental results satisfies practical requirements for numerous applications.

**Figure 15:**
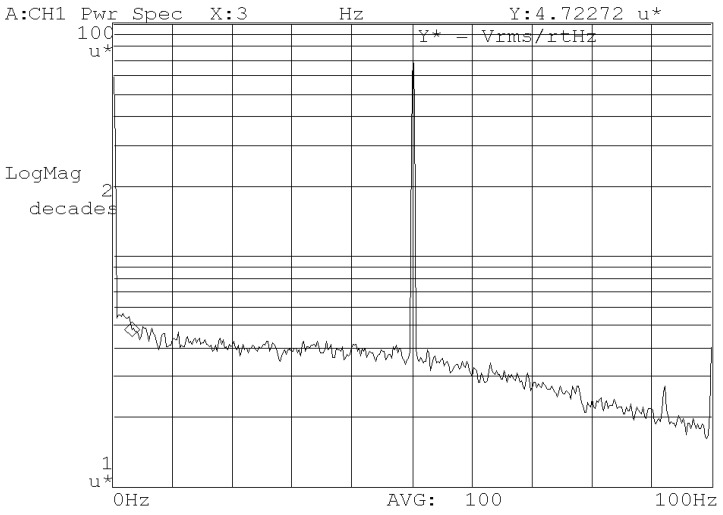
Zero-point output noise.

Noise floor within a bandwidth of 60 Hz at the analog output is measured by a dynamic analyzer, as shown in Figure 15. Only power line interference remains in the measurement band. The 5 μV/√Hz noise floor indicates an equivalent rate noise density no more than 0.0009 °/s/√Hz.

It is also worthwhile to mention that further performance improvement, especially in temperature insensitivity and high density of integration, of the proposed interface ASIC can be achieved by applying different enhancement techniques. As an example, in systematic architecture, a digital closed drive loop has shown good results [10,22]. In this case, extra discrete devices (resistors, capacitors, etc.) on PCB are no more indispensable which are commonly used in analog designs, for higher systematical space efficiency and better compatibility with digital controlling systems in various equipment. Apart from this, it is also advantageous to decrease performance deterioration resulting from temperature drift. From the perspective of high integration demanded in spatial navigation, the capability of triaxial inertial sensing is a positive development direction as presented in [23,24]. So restrained assembly interspace can be taken better advantage of, particularly in miniature devices or military applications.

## 6. Conclusions

In this paper, an interface ASIC for MEMS vibratory gyroscopes is implemented, with a special focus on rapid oscillation and precise driving of the central proof mass in the MEMS sensing element. The use and effectiveness of an incremental zoom ADC are investigated, with remarkable data conversion precision within a low bandwidth, particularly for analog signal detection and processing in gyroscopes and other universal inertial sensor devices. On-chip temperature drift calibration implemented by a digital logic is also crucial for overall systematic robustness to temperature nonidealities.

In order for accurate and stable control of the mechanical sensing element, a self-excited closed drive loop, primarily consisting of a PI-controller and a nonlinear multiplier, has been proposed. This design combines the superiorities in rapid resonance and low-distortion accurate driving of simultaneous square-wave and sinusoidal driving. Switch demodulation is adopted in the sense loop for conciseness. The analog output is obtained after the process of lowpass filtering. After data conversion and on-chip temperature compensation, a digital output is accepted as the final output. The prototype interface ASIC has been fabricated in a standard 0.35 μm CMOS technology. The achieved 2.2 °/h and 200 ms of bias instability and start-up time make the proposed interface ASIC one of the impressive designs among published works.

## Figures and Tables

**Table 1:** Linearity simulation results of the proposed nonlinear multiplier.

in- (V)	*V*_fb_- (V)
0.1	0.103
0.2	0.206
0.3	0.309
0.4	0.411
0.5	0.515
0.6	0.615
0.7	0.720
0.8	0.825
0.9	0.925
1	1.035

## References

[1] Chen F., Li X.X., Kraft M. (2016). Electromechanical sigma-delta modulators (ΣΔM) force feedback interfaces for capacitive MEMS inertial sensors: A review. IEEE Sens. J..

[2] Rombach S., Marx M., Nessler S., De Dorigo D., Maurer M., Manoli Y. (2016). An interface ASIC for MEMS vibratory gyroscopes with a power of 1.6 mW, 92 dB DR and 0.007°/s/$\sqrt{\mathrm{Hz}}$ noise floor over a 40 Hz band. IEEE J. Solid-State Circuits.

[3] Huang F.X., Yin L. (2017). Analysis and design of the system of a total digital Si-gyroscope. Int. J. Mod. Phys. B.

[4] Li X.Y., Hu J.P., Liu X.W. (2018). A high-performance digital interface circuit for a high-Q micro-electromechanical system accelerometer. Micromachines.

[5] Picerno P. (2017). 25 years of lower limb joint kinematics by using inertial and magnetic sensors: A review of methodological approaches. Gait Posture.

[6] Li X.Y., Hu J.P., Chen W.P., Yin L., Liu X.W. (2018). A novel high-precision digital tunneling magnetic resistance-type sensor for the nanosatellites’ space application. Micromachines.

[7] Ahn C.H., Ng E.J., Hong V.A., Yang Y.S., Lee B.J., Flader I., Kenny T.W. (2015). Mode-matching of wineglass mode disk resonator gyroscope in (100) single crystal silicon. J. Microelectromech. Syst..

[8] Maeda D., Ono K., Giner J., Matsumoto M., Kanamaru M., Sekiguchi T., Hayashi M. (2018). MEMS gyroscope with less than 1-deg/h bias instability variation in temperature range from −40 °C to 125 °C. IEEE Sens. J..

[9] Sonmezoglu S., Alper S.E., Akin T. (2014). An automatically mode-matched MEMS gyroscope with wide and tunable bandwidth. J. Microelectromech. Syst..

[10] Qiu B.M., Wang J.W., Li P.H. (2015). Full digital control of hemispherical resonator gyro under force-to-rebalance mode. IEEE Sens. J..

[11] Lee J., Yun S.W., Rhim J. (2016). Design and verification of a digital controller for a 2-piece hemispherical resonator gyroscope. Sensors.

[12] Challoner A.D., Ge H.H., Liu J.Y. Boeing Disc Resonator Gyroscope. Proceedings of the 2014 IEEE/ION Position, Location and Navigation Symposium—PLANS 2014.

[13] Ahn C.H., Shin D.D., Hong V.A., Yang Y.S., Ng E.J., Chen Y.H., Flader I.B., Kenny T.W. Encapsulated disk resonator gyroscope with differential internal electrodes. Proceedings of the 2016 IEEE 29th International Conference on Micro Electro Mechanical Systems (MEMS).

[14] Yoon S., Park U., Rhim J., Yang S.S. (2015). Tactical grade MEMS vibrating ring gyroscope with high shock reliability. Microelectron. Eng..

[15] Chouvion B., McWilliam S., Popov A.A. (2018). Effect of nonlinear electrostatic forces on the dynamic behaviour of a capacitive ring-based Coriolis Vibrating Gyroscope under severe shock. Mech. Syst. Signal Process..

[16] Zhao Y., Zhao J., Wang X., Xia G.M., Shi Q., Qiu A.P., Xu Y.P. (2018). A sub-0.1 degrees/h bias-instability split-mode MEMS gyroscope with CMOS readout circuit. IEEE J. Solid-State Circuits.

[17] Yazdi N., Ayazi F., Najafi K. (1998). Micromachined inertial sensors. Proc. IEEE.

[18] Chae Y., Souri K., Makinwa K.A.A. (2013). A 6.3 μW 20 bit incremental zoom-ADC with 6 ppm INL and 1 μV offset. IEEE J. Solid-State Circuits.

[19] Lv R.S., Chen W.P., Yin L., Fu Q., Liu X.W., Yan J.M. (2018). A closed-loop ΣΔ modulator for micromechanical capacitive sensors. IEICE Electron Express.

[20] Lv R.S., Chen W.P., Liu X.W. (2018). A high-dynamic-range switched-capacitor sigma-delta ADC for digital micromechanical vibration gyroscopes. Micromachines.

[21] He C.H., Zhao Q.C., Huang Q.W., Liu D.C., Yang Z.C., Zhang D.C., Yan G.Z. (2015). A MEMS vibratory gyroscope with real-time mode-matching and robust control for the sense mode. IEEE Sens. J..

[22] Marx M., De Dorigo D., Nessler S., Rombach S., Manoli Y. (2018). A 27 µW 0.06 mm^2^ background resonance frequency tuning circuit based on noise observation for a 1.71 mW CT-ΔΣ MEMS gyroscope readout system with 0.9 °/h bias instability. IEEE J. Solid-State Circuits.

[23] Tan Z.C., Nguyen K., Yan J., Samuels H., Keating S., Crocker P., Clark B. A dual-axis MEMS vibratory gyroscope ASIC with 0.0061 °/s/√Hz noise floor over 480 Hz bandwidth. Proceedings of the 2017 IEEE Asian Solid-State Circuits Conference (A-SSCC).

[24] Balachandran G.K., Petkov V.P., Mayer T., Balslink T. (2016). A 3-axis gyroscope for electronic stability control with continuous self-test. IEEE J. Solid-State Circuits.

